# OsCBL1 mediates rice response to local nitrate signaling: insights into regulatory networks and gene expression

**DOI:** 10.3389/fpls.2024.1418119

**Published:** 2024-09-13

**Authors:** Zhao Hu, Dongchen He, Xiaojue Peng, Jing Yang

**Affiliations:** College of Life Science, Nanchang University, Nanchang, China

**Keywords:** local nitrate signaling, lateral root, OsCBL1, rice (*Oryza sativa* L.), RNAseq

## Abstract

Nitrate is a significant source of nitrogen in soils and also serves as a critical signal for root development. Previous studies have demonstrated that the local nitrate supply promotes lateral root elongation primarily through local nitrate signals, rather than nutritional effects. In this study, we report that Calcineurin B-like protein 1 (OsCBL1) positively regulates local nitrate signaling, thereby triggering lateral root colonization, as revealed by a comparative analysis of the phenotype and whole transcriptome of the knockdown mutant (*OsCBL1*-KD) and the wild-type (WT). In the split-root system, the knockdown of *OsCBL1* was found to inhibit local nitrate-induced lateral root growth. Transcriptome analyses identified 398 differentially expressed genes (DEGs) that were under the control of OsCBL1 and associated with the phenotype of nitrate-induced lateral root colonization. Further analysis revealed that the nitrate transporter/sensor gene *OsNRT1.1B* was up-regulated under Sp-NaNO_3_ conditions compared to Sp-NaCl in WT but not in *OsCBL1*-KD plants. Pathway mapping of DEGs (i.e., genes exhibiting a significant change in expression in the Sp-NaNO_3_ condition compared to the Sp-NaCl condition) revealed a preferential upregulation of genes involved in lignin biosynthesis and a downregulation of genes involved in auxin and salicylic acid signaling. This suggests that OsCBL1 might function as a transmitter within the auxin, salicylic acid signaling, lignin biosynthesis, and nitrate sensor (OsNRT1.1B)-mediated pathways in response to local nitrate signaling. We also identified a transcriptional regulatory network downstream of OsCBL1 in nitrate-rich patches that is centered on several core transcription factors. Our study provides new insights into how plants adapt to an inhomogeneous distribution of nitrogen in the soil.

## 1 Introduction

Nitrogen is an essential macronutrient, the availability of which plays a crucial role in determining plant growth, development and crop productivity (Wang et al., 2018; Gao et al., 2022). However, nitrate, the primary source of nitrogen accessible to plants, is often unevenly distributed in soils (Crawford and Glass, 1998; Smith et al., 2007; Krouk et al., 2010a). In order to compete for nitrate in different soil microenvironments, plants preferentially extend lateral roots (LR) in nitrate-rich areas (Alvarez et al., 2012; Nacry et al., 2013). The phenotype of local nitrate triggering LR proliferation was observed only when plants were supplied with nitrate in a vertical system. This was not seen with other nitrogen sources, confirming the signaling properties of nitrate itself in LR proliferation. Additionally, this phenomenon was also evident in split-root systems, further demonstrating that local nitrate signaling can stimulate LR elongation (Zhang and Forde, 1998; Zhang et al., 1999; Remans et al., 2006; Yan et al., 2014).

In *Arabidopsis*, several genes have been shown to be involved in this process. AtNRT1.1 functions as both a nitrate transporter and sensor (Ho et al., 2009). Mutants of AtNRT1.1 show reduced LR growth in nitrate-rich patches (Remans et al., 2006). The transcription factor AtTCP20 plays a crucial role in nitrate signaling. Insertion mutants of AtTCP20 (*tcp20-2* and *tcp20-4*) exhibit impaired preferential LR growth on heterogeneous media in split-root plates, but normal primary and lateral root growth on homogeneous nitrate media (Guan et al., 2014). Furthermore, the interaction between AtSTOP1 and AtTCP20 co-regulates nitrate signaling under nitrate-deficient conditions by effectively activating AtNRT1.1 expression (Tokizawa et al., 2023). AtNRT1.1 also acts as an auxin transporter, facilitating preferential root colonization of nitrate-rich patches. It inhibits root growth in response to low nitrate levels by modulating auxin traffic, while promoting root growth in response to high nitrate levels (Krouk et al., 2010b; Mounier et al., 2014).

Lignin is an essential component of plant cells, critical for growth and metabolism (Barros et al., 2015). Originating from the phenylpropanoid pathway, lignin supports numerous biological processes throughout plant growth (Brunetti et al., 2018; Vanholme et al., 2019). Recent studies have shown a close correlation between lignin metabolism and root growth. In wheat, the TaANR1 and TaMADS25 complexes positively regulate root development through lignin biosynthesis (Xu, Chen et al., 2023). Additionally, lignin synthesis is involved in the formation of adventitious roots in lotus seedlings (Cheng et al., 2022). The collective findings of these reports indicate that lignin metabolism is closely associated with the growth of plant roots. Additionally, nitrate signaling plays a regulatory role in the growth of plant roots. However, it remains unclear whether there is a correlation between nitrate signaling and lignin synthesis, or whether nitrate signaling regulates plant root growth by modulating lignin metabolism.

Plant root growth is regulated by local and systemic nitrate signals, which can be distinguished but are nevertheless highly interrelated (Ruffel et al., 2011). The local nitrate response is induced in specific tissues, whereas the systemic nitrate response propagates in all cell types (O'Brien et al., 2016). In rice, OsCBL1 has been demonstrated to regulate root growth through nitrate signaling. This conclusion is based on the observation that knockdown of *OsCBL1* does not affect nitrate uptake but does impact the expression of nitrate-responsive genes when the rice root system is cultured under varying nitrate concentrations (Yang et al., 2019). Furthermore, OsCBL1 deficiency renders rice growth more susceptible to nitrate deficiency. However, this is not attributable to a reduction of nitrate *in vivo*, but may be associated with intricate developmental regulatory signals triggered by nitrate signaling (Hu et al., 2021). The collective findings of these reports suggest that OsCBL1 plays a significant role in the rice response system nitrate signaling pathway. However, the specific role of OsCBL1 in local nitrate signaling remains unclear. In this study, we found that OsCBL1 is essential for local nitrate signaling, as demonstrated by the split-root experiment. In the heterogeneous split-root system, local nitrate promotes lateral root elongation but does not affect embryonic crown roots (ECRs) growth in WT plants. However, this phenotype was absent in *OsCBL1*-KD plants, indicating that OsCBL1 positively regulates local nitrate-induced LR colonization. Furthermore, RNA-seq analysis was conducted to elucidate the underlying molecular mechanism, revealing that OsCBL1 likely regulates local nitrate signaling through the nitrate sensor (OsNRT1.1B), lignin biosynthesis and plant hormone signaling pathway, thus triggering LR colonization.

## 2 Materials and methods

### 2.1 Plant materials and growth conditions

The wild-type rice ShijinB and transgenic *OsCBL1*-knockdown (*OsCBL1*-KD) plants used in this study were previously described (Yang et al., 2019; Hu et al., 2021, 2023). To perform the split-root experiments, both WT and *OsCBL1*-KD rice seeds were sterilized with 5% (v/v) NaClO at room temperature for 30 min and then germinated in a dark incubator at 30°C for 2-3 days. After germination, seedlings of similar size were transferred to an 8-L hydroponic box and grown in a growth chamber with a photoperiod of 12h (light)-12h (dark) (~200μ molm^−2^ s^−1^) at 30 °C/28 °C and 70% humidity. After 5 days of seedling growth in H_2_O, radicle and excess embryonic crown roots (ECRs) were pruned, retaining only two similar ECRs without lateral roots. For the heterogeneous split-root, one ECR was exposed to 2 mM NaNO_3_ (Sp-NaNO_3_) and the other ECR to 2 mM NaCl (Sp-NaCl). For the homogenous split-root, both ECRs were incubated in 2 mM NaNO_3_ or 2 mM NaCl. After 3 days of treatment, rice roots were harvested for phenotyping and RNA-seq analysis.

### 2.2 Phenotyping of seedlings

Roots of WT and *OsCBL1*-KD seedlings, grown for 3 days in NaNO_3_ or NaCl (in the split-root system) as previously described, were imaged at high resolution with a Canon EOS 7D digital SLR camera. The lengths of ECRs and LRs were measured using ImageJ. A minimum of six independent biological replicates were analyzed for each rice variety.

### 2.3 RNA isolation and qPCR analysis

Total RNA isolation and RT-qPCR analyses followed the protocol described previously (Hu et al., 2021). Total RNA was isolated using TRNzol Universal (TIANGEN, Cat no. DP424), reverse transcription was conducted using the FastKing RT Kit (TIANGEN, Cat no. KR116), and qPCR assays employed Power SYBR Green Master Mix. qPCR data were analyzed with GraphPad Prism 8. Three biological replicates were obtained for each data point across all genes. The 2^(-ΔΔCt)^ method was used to assess the fold change of gene expression, with OsActin1 as reference gene and normalized against levels in the Sp-NaCl condition. Relative gene expression levels were displayed as the 2^(-ΔCt)^ value with OsActin1 as the reference gene. Associated primers are detailed in **Supplementary Table S1**.

### 2.4 RNA sequencing and data analysis

Roots of WT and *OsCBL1*-KD seedlings grown for 3 days in Sp-NaNO_3_ or Sp-NaCl (using the heterogeneous split-root system as described) were harvested and divided into three biological replicates (each biological replicate comprised 10 - 12 ECRs with a total fresh weight of 0.1 g) for RNA-seq library preparation, resulting in a total of 12 libraries. Using the Illumina platform, the libraries were submitted to novaseq6000 for sequencing. The raw data were filtered using Trimmomatic to eliminate low-quality reads (Bolger et al., 2014). High-quality reads were aligned to the NIP reference genome (ftp://ftp.ensemblgenomes.org/pub/plants/release-44/fasta/oryza_sativa/dna/) using STAR software (Dobin et al., 2013). Following alignment, the raw counts were normalized to the Trimmed Mean of M values (TMM) using RSEM for gene expression quantification (Li and Dewey, 2011). Differential analysis between the two samples was carried out by DESeq2 using raw counts, identifying genes as differential if padj<0.05 and |log2FC|>1. Gene Ontology (GO) enrichment analysis was conducted using the DAVID Bioinformatics online platform (https://david.ncifcrf.gov/content.jsp?file=citation.htm) (Sherman et al., 2022), and the results were visualized using the Bioinformatics online platform (http://www.bioinformatics.com.cn). Kyoto Encyclopedia of Genes and Genomes (KEGG) pathway analysis was performed using TBtools, with data obtained from the KEGG website (https://www.kegg.jp/) (Chen et al., 2023).

### 2.5 TF prediction

Transcription factor (TF) predictions were performed by aligning differentially expressed genes to the rice (Oryza sativa) gene functional annotation database at NCBI (https://www.ncbi.nlm.nih.gov/) and further validated on the RiceTFtarget website (https://cbi.njau.edu.cn/RiceTFtarget/). Target genes for the TFs were then predicted using the RiceTFtarget website, and filtered by overlapping with differentially expressed genes (Zhang et al., 2023). The network visualization was conducted with Cytoscape software. The genes in the red boxes represent key TFs, while the blue circles indicate predicted genes that may be activated or repressed in expression by TFs. The gray lines represent possible transcriptional activation or repression.

### 2.6 Statistical analysis

Experimental data were collected to calculate averages and standard deviation (SD). The number of biological replicates is detailed in the legend of each figure. Statistical significance was determined using Student’s t-test and one-way ANOVA, with a significance level of P < 0.05. All statistical analyses were conducted using GraphPad Prism 8 statistical software.

## 3 Result

### 3.1 OsCBL1 positively regulates the response to local nitrate signaling

**Figure 1:**
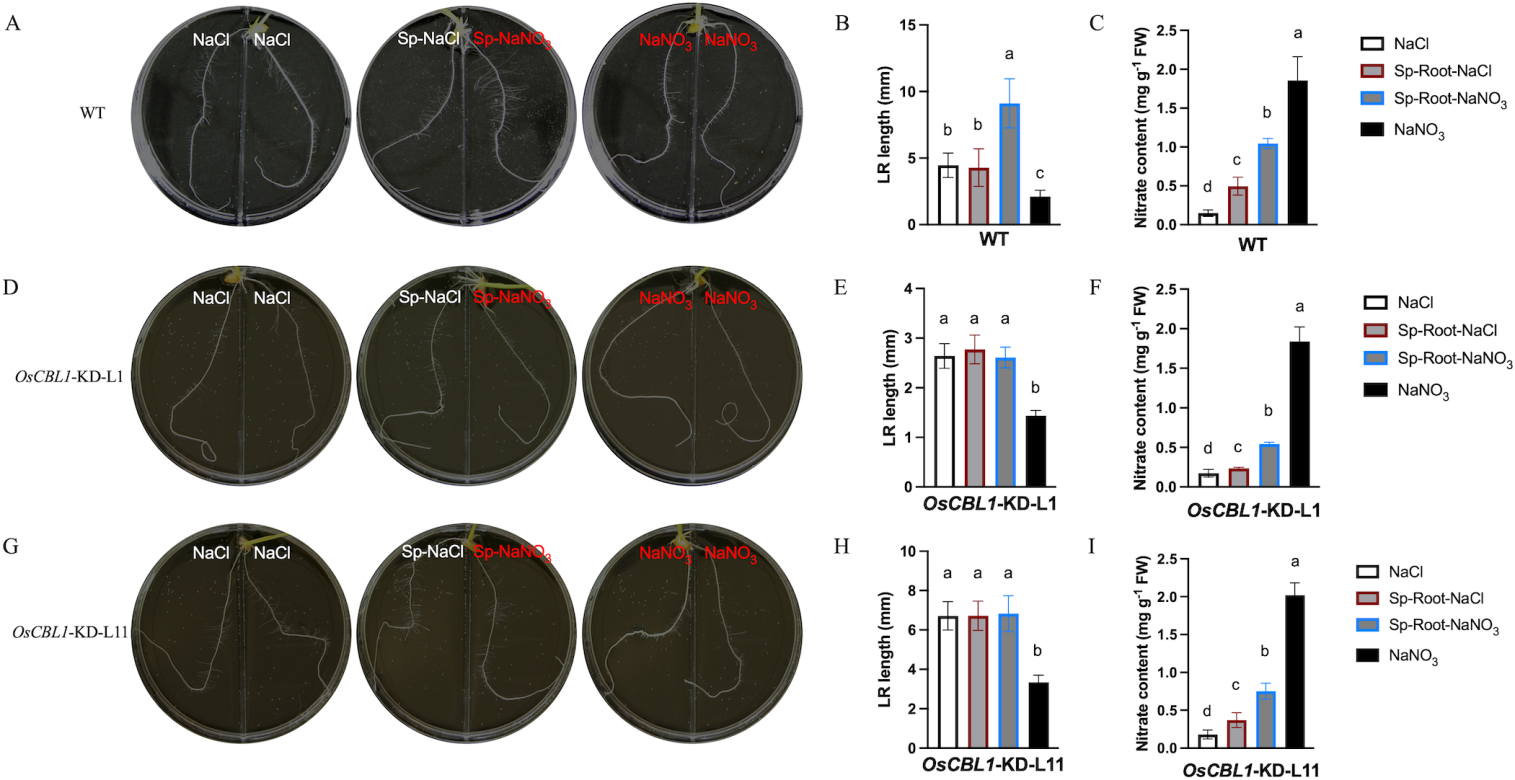
Knockdown of *OsCBL1* impaired the nitrate signaling in the split-root system. The phenotypes of WT **(A)** and *OsCBL1*-KD **(D, G)** on 9 cm diameter split plates in the split-root system. Nitrate rich zones: 2 mM NaNO_3_; nitrate absent zones: 2 mM NaCl. The LR length of WT **(B)** and *OsCBL1*-KD **(E, H)** in the split-root system. n ≥ 6 biologically independent samples. The nitrate content of WT **(C)** and *OsCBL1*-KD **(F, I)** in the split-root system. n = 3 biologically independent samples. The error bars represent ± SD. Different letters above bars indicate statistically significant difference between samples (one way ANOVA, P < 0.05).

Our previous study demonstrated that OsCBL1 functions as a hub in rice response to nitrogen signaling (Yang et al., 2019; Hu et al., 2021, 2023). To further understand the function of OsCBL1 in local nitrate signaling, we analyzed the root architecture of *OsCBL1*-KD and WT plants in the split-root system. The split-root system used to study local nitrate signaling consisted of a heterogeneous split condition in which two embryonic crown roots (ECRs) were placed separately in 2 mM NaNO_3_ (Sp-NaNO_3_) or 2 mM NaCl (Sp-NaCl). Homogenous treatment (two ECRs, both in 2 mM NaNO_3_ or 2 mM NaCl) served as controls. In WT plants, compared to NaCl, Sp-NaCl treatment did not increase the length of the lateral root (LR), whereas Sp-NaNO_3_ treatment significantly increased the length of the LR (**Figures 1A, B**). In comparison to NaNO_3_, both Sp-NaCl and Sp-NaNO_3_ treatments increased the length of the LR in WT plants (**Figure 1B**). Under the heterogeneous split-root condition, local nitrate (Sp-NaNO_3_) increased LR elongation compared to Sp-NaCl in WT plants (**Figure 1B**; **Supplementary Figure S1B**). However, the nitrate content of the roots of WT plants in the four environments was, in descending order, as follows: NaNO_3_ > Sp-NaNO_3_ > Sp-NaCl > NaCl (**Figure 1C**). These results suggest an inconsistency between the length of the LR and the amount of nitrate content (**Figures 1B, C**), and suggesting that LR length is related to nitrate signaling rather than nitrate content. For *OsCBL1*-KD plants, the LR length is longer under Sp-NaCl and Sp-NaNO_3_ conditions than under NaNO_3_ condition, but did not change under NaCl condition (**Figures 1D, E, G, H**). It is crucial to highlight that, in contrast to WT, local nitrate (Sp-NaNO_3_) supply did not result in an increase in LR elongation in *OsCBL1*-KD plants under heterogeneous split-root conditions compared to Sp-NaCl (**Figures 1B, E, H**; **Supplementary Figure S1B**). These findings indicate that OsCBL1 may play a crucial role in rice response to local nitrate signaling.

**Figure 2:**
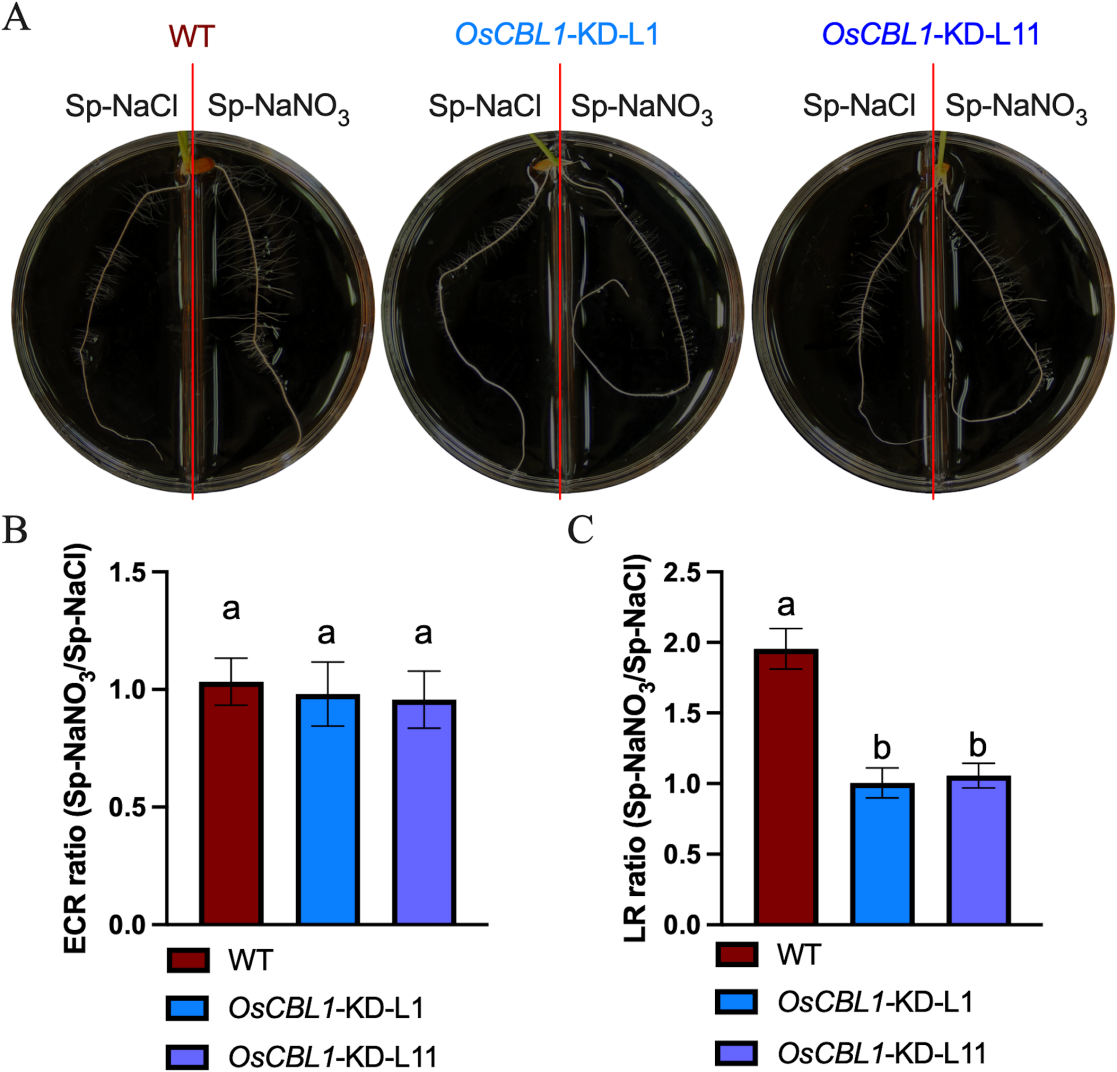
Knockdown of *OsCBL1* inhibits the lateral root growth in nitrate-rich zones in the heterogeneous split-root system. **(A)** The phenotypes of *OsCBL1*-KD and WT on 9 cm diameter split plates. Nitrate rich zones: 2 mM NaNO_3_; nitrate absent zones: 2 mM NaCl. **(B)** The ECR ratio of Sp-NaNO_3_/Sp-NaCl in *OsCBL1*-KD and WT. **(C)** The LR ratio of Sp-NaNO_3_/Sp-NaCl in *OsCBL1*-KD and WT. n ≥ 6 biologically independent samples. The error bars represent ± SD. Different letters above bars indicate statistically significant difference between samples (one way ANOVA, P < 0.05).

Consequently, a detailed analysis was conducted on the phenotypes of WT and *OsCBL1*-KD plants in a heterogeneous split-root system. For WT plants, ECR lengths were 96.46 mm under Sp-NaNO_3_ and 93.77 mm under Sp-NaCl (**Figure 2A**; **Supplementary Figure S1A**). LR lengths were 9.10 mm under Sp-NaNO_3_ and 4.28 mm under Sp-NaCl (**Figure 2A**; **Supplementary Figure S1B**). The ECR ratio between Sp-NaNO_3_ and Sp-NaCl was 1.03, and the LR length ratio between Sp-NaNO_3_ and Sp-NaCl was 1.95 (**Figures 2B, C**). These results indicated that local nitrate promotes lateral root elongation but does not affect ECR growth in the split-root system. In the heterogeneous split-root system, ECR length for *OsCBL1*-KD-L1 or L11 were 97.69 (L1) mm, 117.58 (L11) mm under Sp-NaNO_3_ and 94.88 (L1) mm, 110.95 (L11) mm under Sp-NaCl (**Figure 2A**; **Supplementary Figure S1A**). However, LR lengths of *OsCBL1*-KD were 2.97 (L1) mm, 6.83 (L11) mm under Sp-NaNO_3_, significantly lower than those of WT (**Figure 2A**; **Supplementary Figure S1B**). Consequently, the ECR ratio between Sp-NaNO_3_ and Sp-NaCl is 0.98 (L1) and 0.95 (L11), which is not significantly different from WT (**Figure 2B**). The lateral root length ratio between Sp-NaNO_3_ and Sp-NaCl was 1.00 (L1), and 1.05 (L11) respectively, which were significantly lower than WT (**Figure 2C**). This suggests that local nitrate promotion of LR elongation depends on OsCBL1.

A previous study found that treating the entire root system with NaNO_3_ increased nitrate content in both WT and *OsCBL1*-KD rice roots, as well as the length of ECRs and LR (Yang et al., 2019). However, in the heterogeneous split-root system, compared to Sp-NaCl, Sp-NaNO_3_ treatment increased nitrate content in WT rice roots and promoted LR growth, but did not affect ECR growth (**Supplementary Figure S1**, **S2**). Meantime, the nitrate content ratio (Sp-NaNO_3_/Sp-NaCl) did not exhibit a significant difference between WT and *OsCBL1*-KD (**Supplementary Figure S2B**). Nevertheless, the phenotype of nitrate trigger LR growth in the Sp-NaNO_3_ zone was not observed in *OsCBL1*-KD plants compared to the Sp-NaCl zone. This suggests OsCBL1 mediates LR elongation promotion by nitrate through local signaling in the heterogeneous split-root system.

### 3.2 Identification of differentially expressed genes controlled by OsCBL1 in response to local nitrate signaling

**Figure 3:**
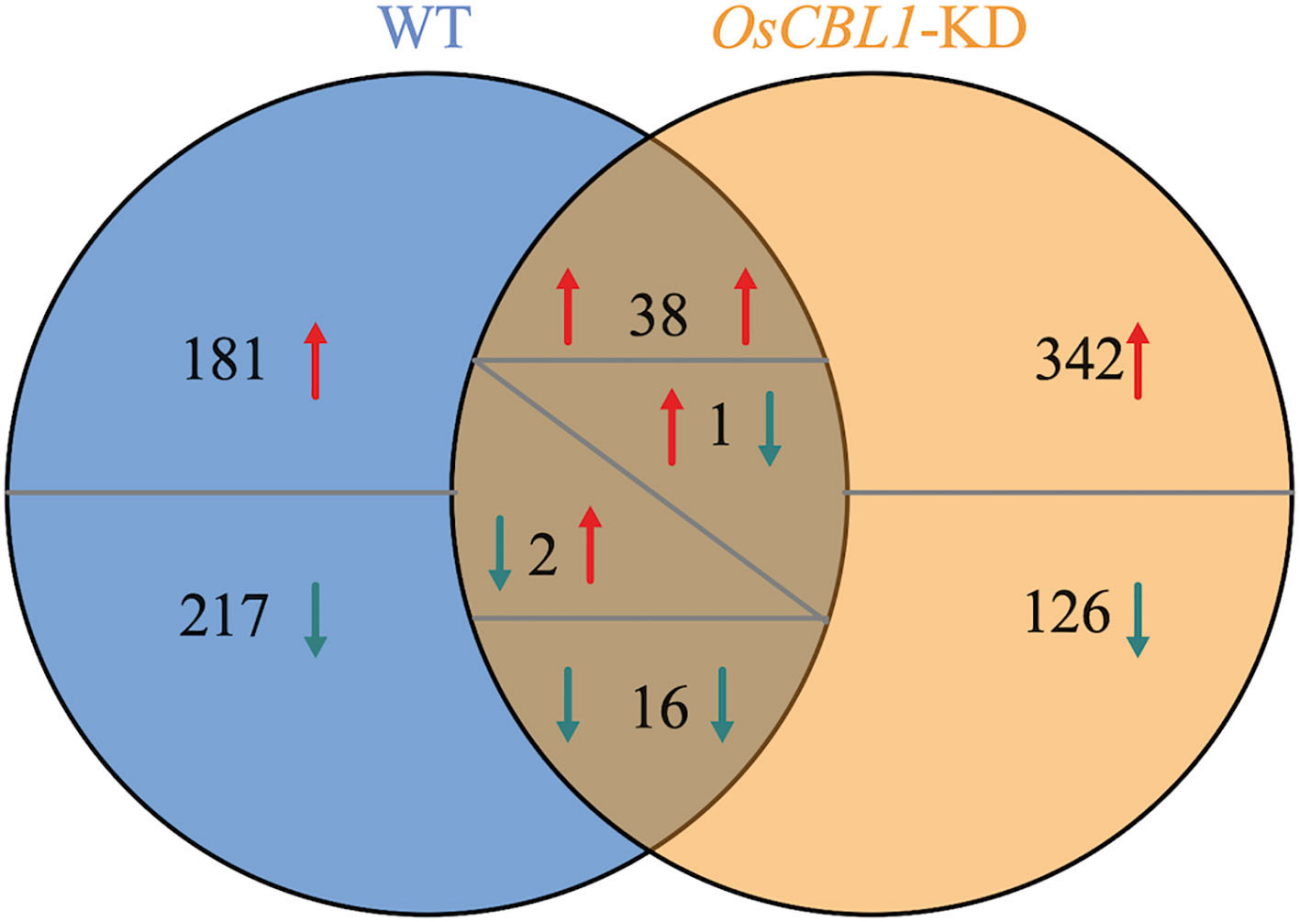
Venn diagram illustrates the number of DEGs in WT and *OsCBL1*-KD in the split-root system. Red arrows indicate up-regulated expression and green arrows indicate down-regulated expression. When a number has arrows on both the left and right side, the left arrow represents the change in gene expression in WT and the right arrow represents the change in gene expression in *OsCBL1*-KD.

Transcriptome analysis was performed to investigate the underlying mechanism of OsCBL1 involved in the regulation of LR elongation by local nitrate signaling. Transcriptome comparisons revealed that a total of 455 differentially expressed genes (DEGs) were identified in WT plants between Sp-NaCl and Sp-NaNO_3_, of which 220 DEGs were up-regulated in the presence of nitrate and 235 DEGs were down-regulated (**Figure 3**). 398 (217 + 181) genes were differentially expressed exclusively in WT plants (**Figure 3**), which represent an OsCBL1-dependent pathway of increasing lateral root growth in nitrate-rich zones. In *OsCBL1*-KD plants, 525 DEGs were identified, of which 382 DEGs were up-regulated and 143 DEGs were down-regulated (**Figure 3**). 54 (16 + 38) genes overlap between WT and *OsCBL1*-KD (**Figure 3**), possibly reflecting an OsCBL1-independent regulatory network of lateral root growth in response to local nitrate signaling. Additionally, 468 (342 + 126) unique DEGs were identified in *OsCBL1*-KD plants (**Figure 3**), which were controlled by OsCBL1 but could not achieve the promotion of LR prolongation by local nitrate signaling.

### 3.3 GO analysis of OsCBL1-dependent/independent-DEGs

**Figure 4:**
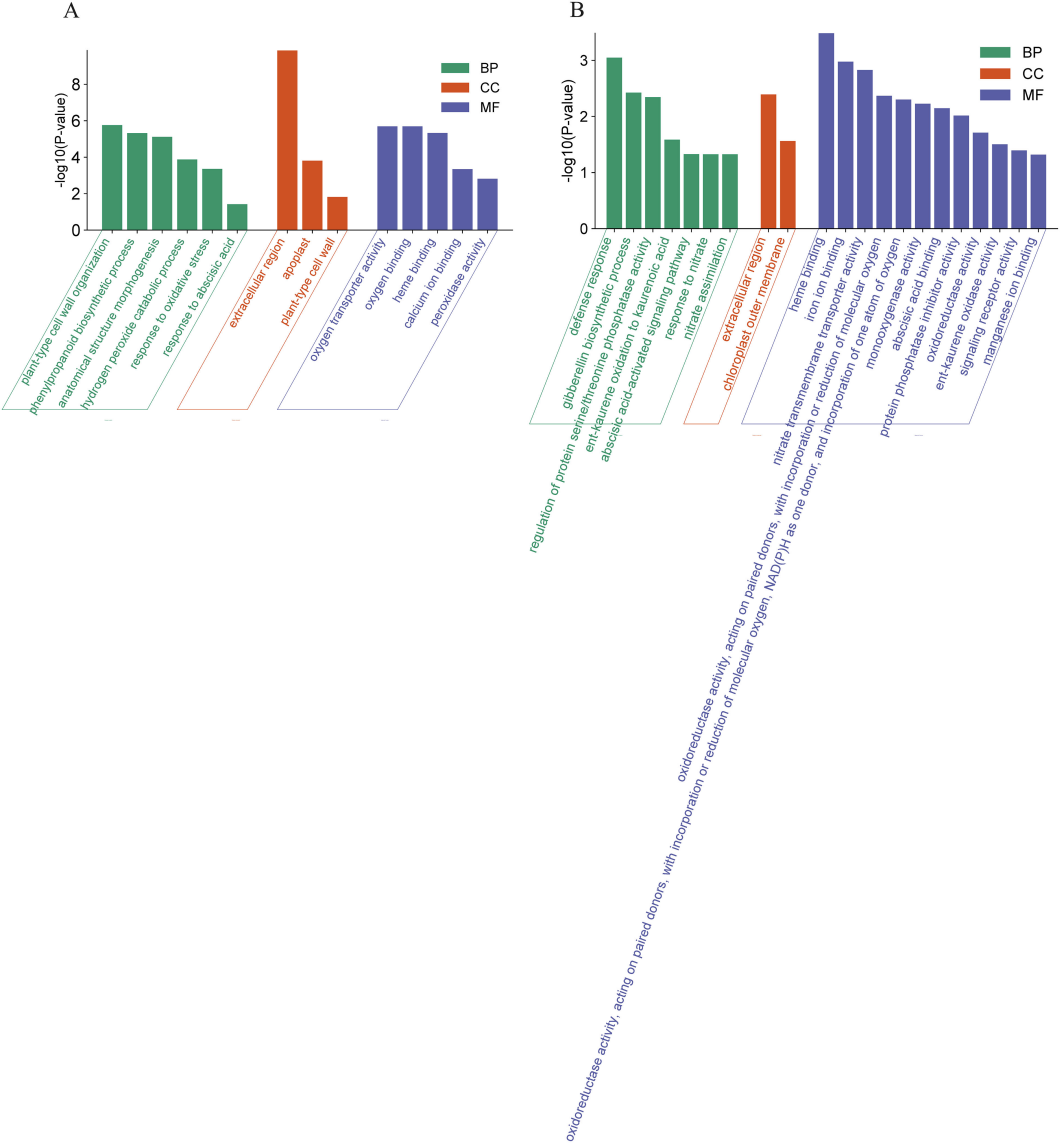
GO analysis of DEGs in WT. **(A)** GO analysis of the up-regulated DEGs in WT. **(B)** GO analysis of the down-regulated DEGs in WT. BP, Biological process; MF, Molecular function; CC, Cell component.

The DEGs in WT represent an OsCBL1-dependent/independent pathway that regulates lateral root growth in response to local nitrate signaling. Therefore, we investigated the function of these DEGs (398 + 54) based on GO classifications (**Figure 4**). For up-regulated DEGs, a total of 14 GO terms (5 Molecular function, 3 Cell component and 6 Biological process) pathways were enriched (**Figure 4A**). In the “Molecular function” domain, up-regulated DEGs were mainly enriched in “oxygen transporter activity”, “oxygen binding” and “heme binding”. In the “Cell component” domain, “extracellular region” was the top annotation, followed by “apoplast” and “plant-type cell wall”. In the “Biological process” domain, “plant-type cell wall organization” and “phenylpropanoid biosynthetic process” were the top annotations, followed by “anatomical structure morphogenesis” and “hydrogen peroxide catabolic process” (**Figure 4A**). For down-regulated DEGs, a total of 21 GO terms (12 Molecular function, 2 Cell component and 7 Biological process) pathways were enriched (**Figure 4B**). In the “Molecular function” domain, the down-regulated DEGs were mainly enriched in “heme binding”, “iron ion binding”, and “nitrate transmembrane transporter activity”. In the “Cell component” domain, the down-regulated DEGs were mainly enriched in “extracellular region” and “chloroplast outer membrane”. In the “Biological process” domain, “defense response” was the top annotation, followed by “gibberellin biosynthetic process”, “regulation of protein serine/threonine phosphatase activity” and “ent-kaurene oxidation to kaurenoic acid” (**Figure 4B**).

In addition, we investigated the functions of DEGs (324 + 126) exclusively altered in *OsCBL1*-KD plants based on the GO classifications (**Supplementary Figure S3**). The major GO pathway enrichment differed from that observed for OsCBL1-dependent DEGs (exclusive to WT) (**Supplementary Figure S4**), indicating that knockdown of *OsCBL1* severely disrupts the functional network of OsCBL1-dependent DEGs, thereby affecting the network of OsCBL1-dependent DEGs and local nitrate signaling, which promotes lateral root elongation.

### 3.4 KEGG analysis of OsCBL1 dependent-DEGs

**Figure 5:**
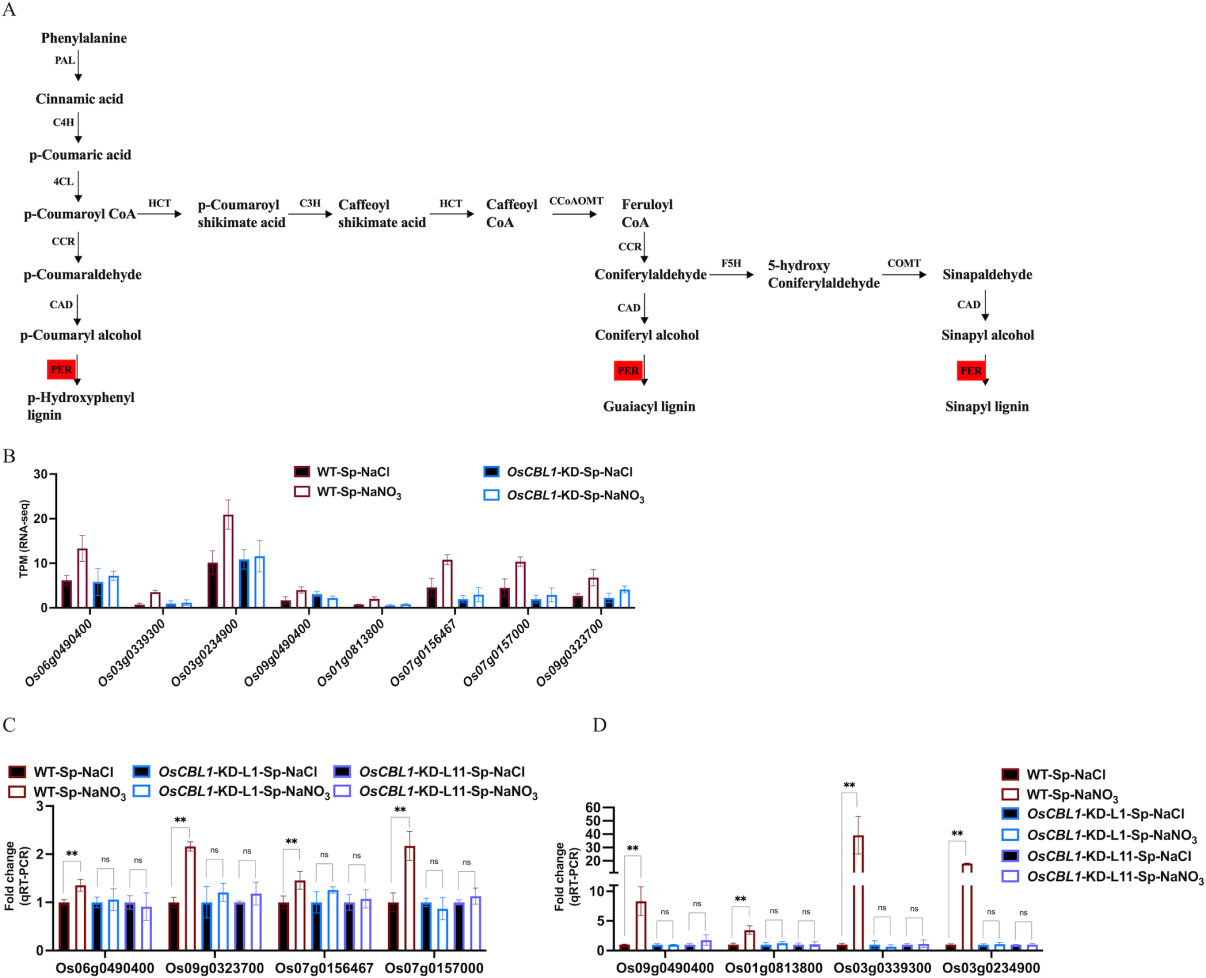
KEGG pathway of phenylpropanoid biosynthetic and the expression analysis of genes related to lignin biosynthesis. **(A)** KEGG analysis of phenylpropanoid biosynthetic pathway. Red markers indicate pathways involved in genes in **Figure 5B**. PAL, phenylalanine ammonia lyase; C4H, cinnamate 4-hydroxylase; 4CL, 4-coumarate: CoA ligase; HCT, hydroxycinnamoyl CoA shikimate hydroxycinnamoyl transferase; CCR, cinnamoyl-CoA reductase; CAD, cinnamyl alcohol dehydrogenase; C3H, p-coumarate 3-hydroxylase; CCoAOMT, caffeoyl-CoA O-methyltransferase; COMT, caffeic acid O-methyltransferase; F5H, ferulate 5-hydroxylase; PER, peroxidase. **(B)** The expression of lignin biosynthesis related genes in RNA-seq. **(C, D)**, qRT-PCR validation of transcriptome data for lignin biosynthesis related genes. n =3 biologically independent samples. The error bars represent ± SD. **p < 0.01 compared to the Sp-NaCl (Student’s t-test). NS indicates no significant difference.

**Figure 6:**
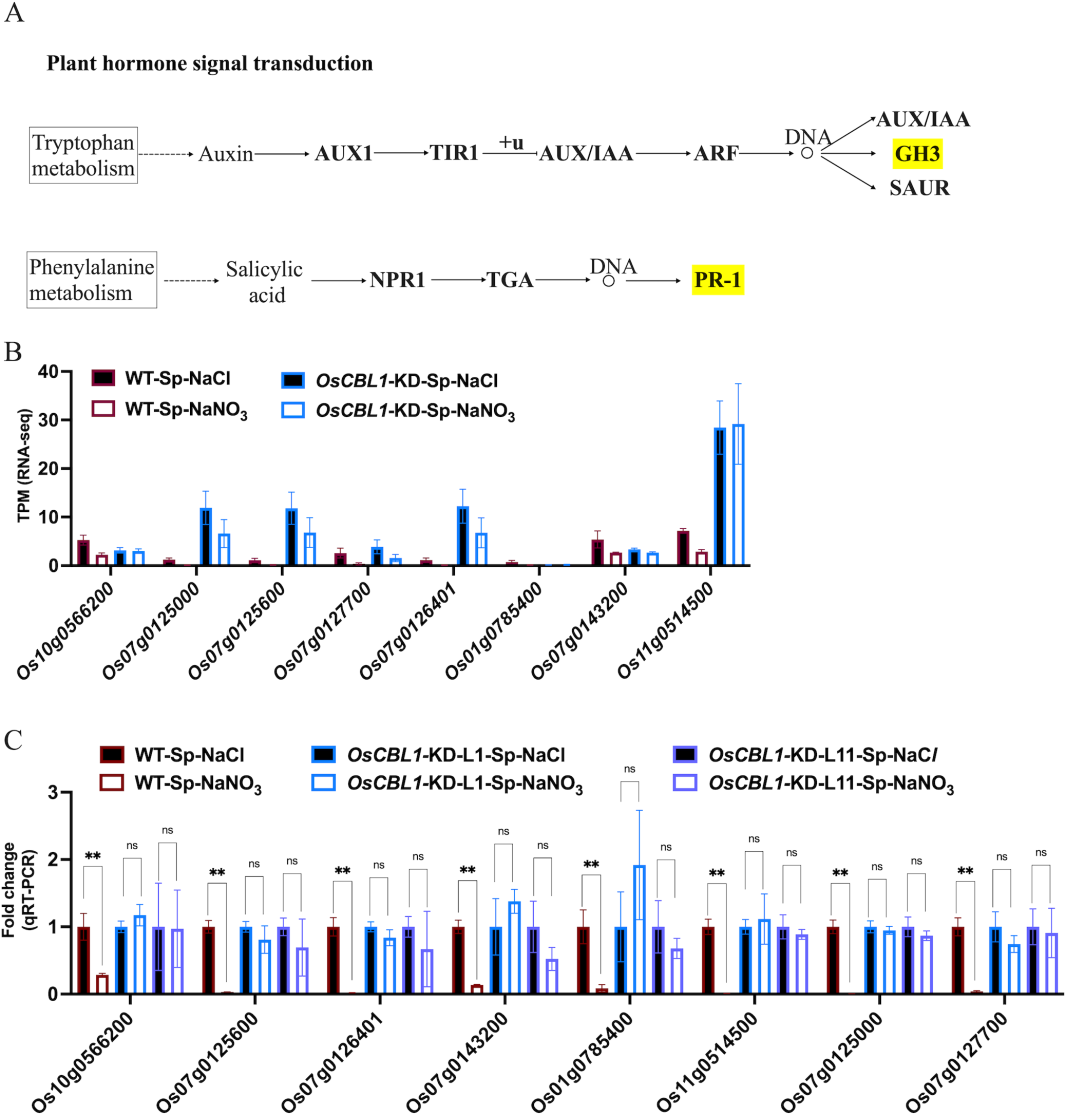
KEGG pathway of plant hormone signal transduction and the expression analysis of genes related to hormone signal. **(A)** KEGG analysis of hormone signal transduction pathway. Yellow markers indicate pathways involved in genes in **Figure 6B**. “+u” stands for ubiquitination. “DNA and circle” refers to a complex of transcription factors bound to DNA. **(B)** The expression of hormone signal related genes in RNA-seq. **(C)** qRT-PCR validation of transcriptome data for hormone signal related genes. n =3 biologically independent samples. The error bars represent ± SD. **p < 0.01 compared to the Sp-NaCl (Student’s t-test). NS indicates no significant difference.

OsCBL1-dependent DEGs (181 + 217), differentially expressed only in WT (**Figure 3**) and associated with the phenotype, were the main cause of local nitrate signaling induced lateral root growth. To further investigate the pathways associated with these DEGs, a pathway-based analysis was performed using the KEGG pathway database (**Supplementary Figure S5**). The up-regulated DEGs were predominantly enriched in phenylpropanoid biosynthesis pathways (**Supplementary Figure S5A**). Among the up-regulated genes in DEGs, eight key genes (*Os06g0490400* (OsPRX80), *Os03g0339300* (OsPRX41), *Os03g0234900* (OsPRX39), *Os09g0490400* (OsBGlu29), *Os01g0813800* (Os1BGlu3), *Os07g0156467*, *Os07g0157000* (OsPRX7), *Os09g0323700* (OsPRX121)) were identified within the phenylpropanoid biosynthetic pathway, involved in the peroxidase (PER) pathway (red marked sections in **Figure 5A**) of lignin biosynthesis (**Figure 5A**), suggesting that local nitrate signaling can modulate lignin biosynthesis. Lignin has been shown to be a positive regulator of lateral root growth (Xu et al., 2023). Compared to Sp-NaCl, genes linked to lignin biosynthesis were significantly up-regulated in Sp-NaNO_3_ in WT plants, a trend not seen in *OsCBL1*-KD plants, confirmed by RT-qPCR (**Figures 5B–D**). These results suggest that OsCBL1 may regulate LR growth promotion in response to local nitrate signaling through the lignin biosynthesis pathway. The down-regulated DEGs were primarily enriched in plant hormone signal transduction pathway (**Supplementary Figure S5B**). Among the down-regulated DEGs, *Os01g0785400* (OsGH3.1) was identified in the GH3 family of the auxin signaling pathway, while *Os10g0566200*, *Os07g0125000* (OsPR1b), *Os07g0125600*, *Os07g0127700*, *Os07g0126401*, *Os07g0143200* (OsPIL14) and *Os11g0514500* were identified within the pathogenesis-related (PR-1) pathway of the salicylic acid (SA) signaling transduction pathway (**Figure 6A**). Compared to Sp-NaCl, these genes were significantly down-regulated in Sp-NaNO_3_ in WT plants, a pattern not observed in *OsCBL1*-KD plants (**Figures 6B, C**). Further analysis revealed that *OsGH3.1* encodes an indole-3-acetic acid-amido synthetase gene involved in the auxin pathway (**Figure 6A**). The GH3 family is reported to negatively regulate LR growth via the auxin pathway (Wang et al., 2023). Given the low expression of *OsGH3.1* in WT under Sp-NaNO_3_, we propose that OsCBL1’s regulation of LR growth in response to local nitrate signaling may also involve the auxin pathway.

### 3.5 Role of OsCBL1 in transcriptional regulatory network in response to local nitrate signaling

**Figure 7:**
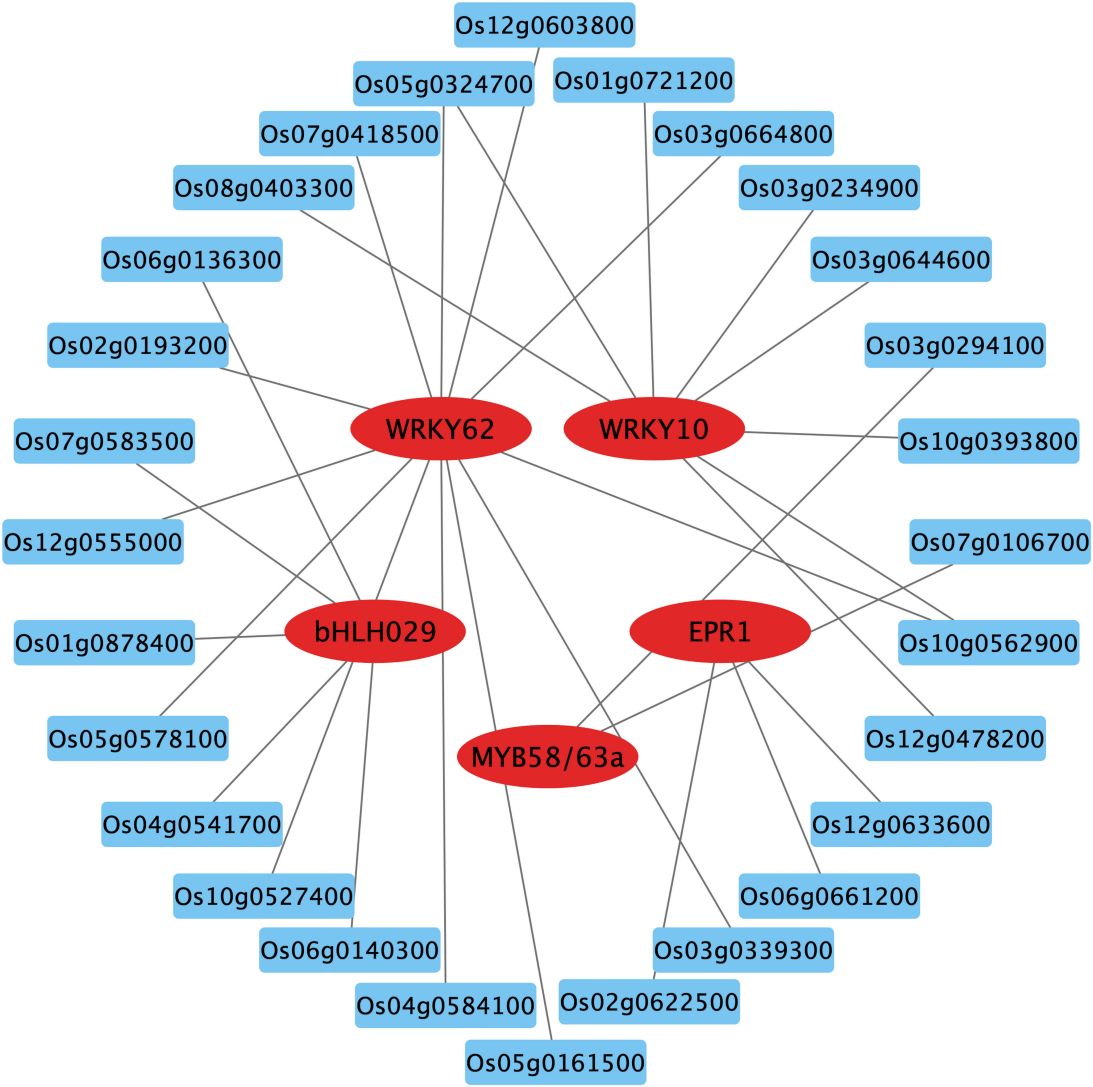
The key TFs and prediction network composed of OsCBL1-dependent DEGs. The genes in the red circles represent key TFs, while the blue boxes indicate predicted genes that may be activated or repressed in expression by TFs. The gray lines represent possible transcriptional activation or repression.

Transcription factors (TFs) are key players in the response to nitrate signaling as they coordinate the expression of downstream genes (Varala et al., 2018). By aligning with the rice gene functional annotation database and validating in the RiceTFtarget website, some key TF family members were identified among the DEGs in WT. These transcription factors include MYB, WRKY and bHLH, which were down-regulated in nitrate-rich zones compared to nitrate-absent zones (**Figure 7**; **Supplementary Figure S6**). The predicted TF network from RiceTFtarget (Zhang et al., 2023) identified 5 core TFs potentially interacting (possible transcriptional activation or repression) with multiple genes among OsCBL1-dependent DEGs (**Figure 7**; **Supplementary Table S2**). These TFs include *Os01g0186000* (OsWRKY10), *Os09g0417800* (WRKY62), *Os02g0695200* (OsMYB58/63a), Os06g0728700 (OsEPR1) and *Os02g0221100* (OsbHLH029), all of which exhibited downregulation in response to local nitrate (under Sp-NaNO₃ conditions) (**Supplementary Figure S6A**). Furthermore, qPCR was performed to ascertain the relative expression levels of *OsWRKY10*, *OsMYB58/63a*, *OsEPR1* and *OsbHLH029* in Sp-NaCl and Sp-NaNO_3_ treatments. The results demonstrated that, in WT plants, the expression of these genes was significantly lower in the Sp-NaNO_3_ treatment compared to Sp-NaCl. However, this pattern was not observed in *OsCBL1*-KD plants (**Supplementary Figure S6B**). This suggests that local nitrate signaling may be mediated through OsCBL1 to OsWRKY10, OsMYB58/63a, OsEPR1 and OsbHLH029 influencing lateral root growth.

OsWRKY62 is induced to be expressed by Salicylic acid (SA) and is involved in the SA response pathway (Ryu et al., 2006). Based on the KEGG analysis, we found that some DEGs were enriched in the SA signaling pathway (**Figure 6**). Further analysis revealed that *OsPR1b* encodes a pathogenesis-related protein involved in the SA signaling pathway (**Figure 6**). Combined with the lower expression of *OsPR1b* and *OsWRKY62* in WT under Sp-NaNO_3_ conditions compared to Sp-NaCl, leading us to propose that OsCBL1 may regulate LR growth in response to local nitrate signaling through the SA pathway.

### 3.6 The effect of OsCBL1 on the nitrate sensor protein *OsNRT1.1B*

**Figure 8:**
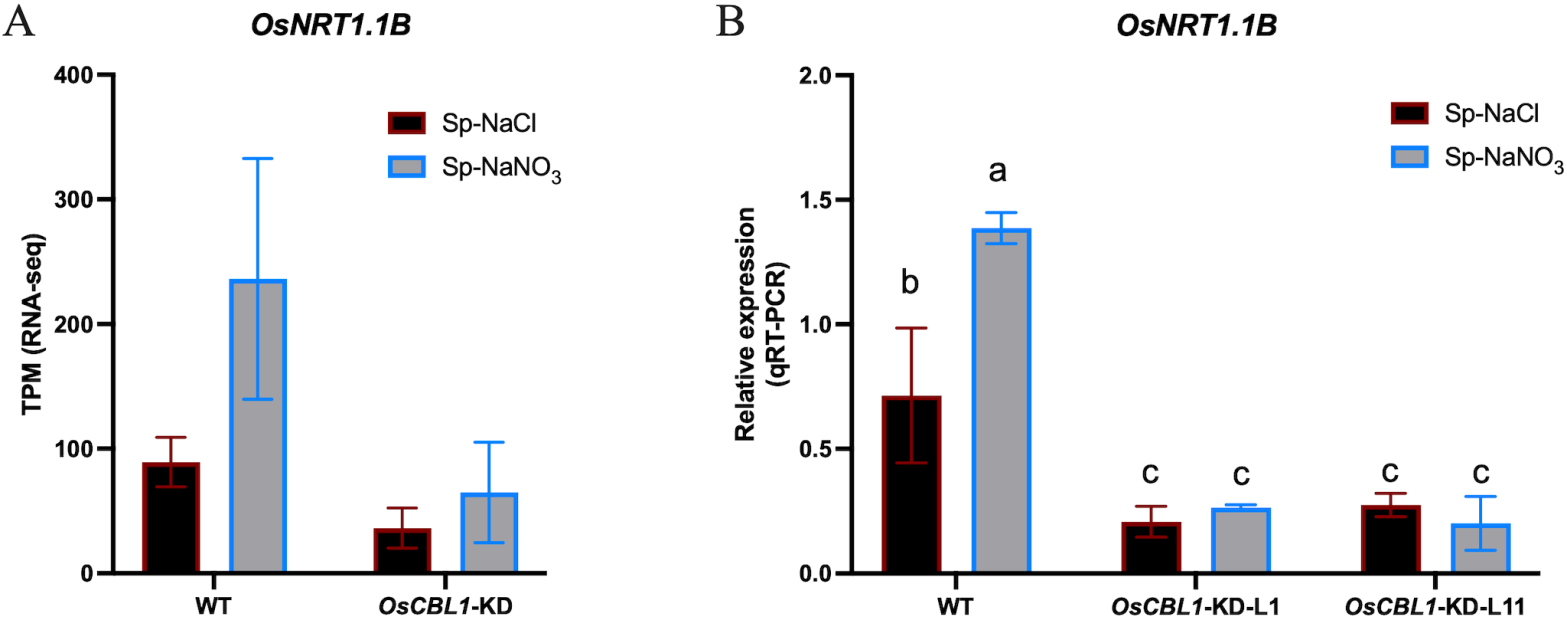
The expression of *OsNRT1.1B* in WT and *OsCBL1*-KD pants. **(A)** The expression of *OsNRT1.1B* in RNA-seq. **(B)** qRT-PCR validation of transcriptome data for *OsNRT1.1B*. n =3 biologically independent samples. The error bars represent ± SD. Different letters above bars indicate statistically significant difference between samples (one way ANOVA, P < 0.05).

Nitrate signaling can be sensed by nitrate sensors (O'Brien et al., 2016; Bellegarde et al., 2017). AtNRT1.1 functions as a nitrate sensor, leading to increased rates of LR elongation through local nitrate signaling. *nrt1.1* mutants show significantly reduced LR colonization of the nitrate-rich patches in the split-root system (Tokizawa et al., 2023). Using RNA-seq, we found that *OsNRT1.1B* (*Os10g0554200*) was present in OsCBL1-dependent differentially expressed genes (**Supplementary Figure S7**). OsNRT1.1B is a functional homologue of AtNRT1.1 and acts as a transceptor, sensing nitrate signals and activating the nitrate response (Hu et al., 2015). The expression of *OsNRT1.1B* was up-regulated under Sp-NaNO_3_ conditions compared to Sp-NaCl in WT but not in *OsCBL1*-KD plants (**Figure 8A**, **Supplementary Figure S7**), as confirmed by RT-qPCR (**Figure 8B**). Considering that OsNRT1.1B is also a nitrate sensor, we suggest that the function of OsCBL1 in rice response to local nitrate signaling may be related to OsNRT1.1B.

## 4 Discussion

The plant responds to the detection of a nitrate-rich patches in the root environment by initiating a crucial developmental response that results in preferential LR growth in the nitrate-rich zone (Alvarez et al., 2012; Nacry et al., 2013). This response is primarily driven by specific local nitrate signaling rather than a nutritional effect, as demonstrated by both vertical and split-root systems (Zhang and Forde, 1998; Zhang et al., 1999; Remans et al., 2006; Yan et al., 2014). Our previous study showed that OsCBL1 plays a crucial role in the nitrogen signaling pathway in rice (Yang et al., 2019; Hu et al., 2021, 2023). To investigate the function of OsCBL1 in local nitrate signaling, we examined the growth phenotype of *OsCBL1*-KD and WT in the split-root system. We discovered that OsCBL1 has a regulatory function in the growth of lateral roots, and specifically, knockdown of *OsCBL1* results in a significant hindrance of lateral root development in Sp-NaNO_3_ zones compared to Sp-NaCl zones (**Figures 1**, **2**). This finding offers new insights into plant responses to local nitrate signaling.

The systemic nitrate response is a phenomenon that occurs in all cell types (O'Brien et al., 2016). Comparisons between homogeneous and heterogeneous split-root experiments are commonly used to analyze systemic nitrate signals (Remans et al., 2006; Ruffel et al., 2011; Chu et al., 2021). There are actually two different systemic nitrate signals, systemic N-demand signals and systemic N-supply signals (Ruffel et al., 2011). Based on the previously reported experiments (Chu et al., 2021), we analyzed the LR ratio of Sp-NaNO_3_/NaNO_3_ and Sp-NaCl/NaCl in WT and *OsCBL1*-KD plants. The results showed that the knockdown of *OsCBL1* significantly decreased the LR ratio of Sp-NaNO_3_/NaNO_3_ compared to WT, whereas the LR ratio of Sp-NaCl/NaCl was not different between WT and *OsCBL1*-KD plants (**Supplementary Figure S8**). Considering the result that the LR ratio of Sp-NaNO_3_/Sp-NaCl in *OsCBL1*-KD were significantly lower than WT (**Figure 2B**), indicating that knockdown of *OsCBL1* impaired the response of rice to systemic N demand signals, but had no significant effects on the systemic N-supply signals. In contrast to the systemic nitrate signal, the local nitrate signaling is induced in specific tissues (O'Brien et al., 2016). Plant roots are able to perceive their surrounding environment, enhance their uptake/assimilation systems, and proliferate specifically in nutrient-rich zones (local signaling) (Ruffel et al., 2011). Furthermore, local nitrate supply can induce LR growth (Zhang and Forde, 1998; Zhang et al., 1999; Remans et al., 2006; Yan et al., 2014). In this study, the local nitrate (Sp-NaNO_3_) can promote the growth of LR in WT compared to Sp-NaCl (**Figure 2**). Nevertheless, the local nitrate (Sp-NaNO_3_) is unable to trigger LR growth in *OsCBL1*-KD plants compared to Sp-NaCl (**Figure 2**). These results suggest that OsCBL1 is involved not only in systemic nitrate signaling but also in local nitrate signaling. Therefore, our findings led us to propose an extension to the current model, suggesting that OsCBL1-mediated regulation of root architecture is under the control of a dual signaling pathway, comprising a local and a systemic signaling component.

OsCBL1 has been shown to be involved in nitrate signaling but does not affect nitrate content/uptake when the entire rice root system is incubated under varying concentrations of nitrate (Yang et al., 2019). In this study, we also examined the nitrate content in rice roots under heterogeneous and homogenous split-root systems, respectively. In the homogenous split-root system, knockdown of *OsCBL1* had no effect on nitrate content (**Figures 1C, F, G**). This finding is consistent with that previously reported (Yang et al., 2019). This suggests that the split-root experiment does not affect the relationship between OsCBL1 and nitrate content under homogeneous nitrate treatment conditions. In heterogeneous conditions, the roots of both WT and *OsCBL1*-KD in the Sp-NaNO_3_ condition exhibited a higher nitrate content than in the Sp-NaCl condition, whereas the nitrate ratio (Sp-NaNO_3_/Sp-NaCl) was not significantly different between WT and *OsCBL1*-KD (**Supplementary Figure S2**). Nevertheless, the *OsCBL1*-KD plants exhibited lower nitrate levels than the WT (**Supplementary Figure S2A**). This phenomenon may be caused by the heterogeneous split-root treatment. In addition, given the abundance of DEGs between *OsCBL1*-KD and WT, it is hypothesized that these DEGs may be associated with the observed difference in nitrate content between *OsCBL1*-KD and WT. However, further investigation is necessary to substantiate this hypothesis.

Nitrate signals are sensed by nitrate sensors and transmitted to downstream components (O'Brien et al., 2016; Bellegarde et al., 2017). AtNRT1.1 plays a pivotal role in the nitrate signaling pathway, functioning as a nitrate sensor. Mutants of AtNRT1.1 exhibited significantly reduced LR colonization in nitrate-rich patches, resulting in diminished capacity for efficient utilization of this localized nutrient resource (Remans et al., 2006). In rice, OsNRT1.1B, a nitrate sensor, has been shown to mediate nitrate signal transduction (Hu et al., 2015). Moreover, higher expression of *OsNRT1.1B* in nitrate-rich zones compared to nitrate-absent zones was observed in WT plants (**Figure 8**, **Supplementary Figure S7**). This suggests that the OsNRT1.1B-mediated signaling pathway may also play an important role in local nitrate signaling. Nitrate treatment can cause intracellular calcium ion fluctuations (Riveras et al., 2015; Liu et al., 2017). The Calcineurin B-like protein (CBL) family is a class of calcium ion sensors specific to plants that decode calcium signals (Tang et al., 2020). Thus, CBL may be located in the early response in nitrate signaling. Given that CBL proteins do not possess transcription factor activity, it can be postulated that this regulatory process occurs via the mediation of other proteins (such as nitrate sensor, transcriptomes, etc.). In *Arabidopsis*, AtCIPK23, which interacted with AtCBL9, is known to regulate the plant responses to nitrate signaling, notably through its interaction with AtNRT1.1, which it phosphorylates at Thr101 (Ho et al., 2009). Despite these interactions, *Atcipk23* mutants did not affect lateral root growth in the split-root system (Tokizawa et al., 2023). In this study, we found that OsCBL1 plays a crucial role in rice response to local nitrate signaling. Knockdown of *OsCBL1* markedly reduced LR colonization in nitrate-rich patches compared to nitrate-absent zones (**Figure 2**; **Supplementary Figure S1**) in heterogeneous split-root system. In addition, the expression of *OsNRT1.1B* did not vary between nitrate-rich zone and nitrate-absent zone in *OsCBL1*-KD plants (**Figure 8**). This indicates that OsCBL1 regulation of local nitrate signaling in rice may be related to the OsNRT1.1B-mediated pathway, but further research is needed to confirm this relationship. Interestingly, in addition to OsCBL1’s ability to regulate the expression of *OsNRT1.1B* between Sp-NaCl and Sp-NaNO_3_ in the same plant; knockdown of *OsCBL1* also inhibited the expression of *OsNRT1.1B* compared to WT (**Figure 8**). Nevertheless, the molecular mechanism underlying the regulation of *OsNRT1.1B* transcription by OsCBL1 remains elusive, representing a compelling avenue for further investigation.

Lignin, an essential component of plant cells during growth and metabolism (Barros et al., 2015), has been documented to regulate plant root growth (Xu, Chen et al., 2023). However, the impact of nitrate signaling on lignin synthesis remains to be elucidated. Our findings reveal that genes related to lignin synthesis were upregulated in nitrate-rich zones compared to the nitrate-absent zones in WT plants (**Figure 5**), indicating that lignin synthesis is influenced by local nitrate signaling. Conversely, the expression of genes related to lignin synthesis did not change between the nitrate-rich zones and the nitrate-absent zones in the *OsCBL1*-KD plants (**Figures 5B–D**). This suggests that local nitrate signals may be transmitted through OsCBL1 to lignin, thus influence lateral root growth.

Phytohormones play a pivotal role in the regulatory network responding to local nitrate signaling in plants (Bellegarde et al., 2017). KEGG analysis revealed that certain DEGs were enriched in auxin and SA signaling pathways (**Supplementary Figure S5**; **Figure 6**). In WT plants, genes related to auxin and SA pathways were down-regulated in nitrate-rich zones compare to nitrate-absent zones (**Figure 6**). However, the expression levels of genes related to auxin and SA pathways did not change between the nitrate-rich and the nitrate-absent zones in the *OsCBL1*-KD plants (**Figure 6**). These findings indicate that OsCBL1 serves as a critical node in hormone signaling under local nitrate conditions, shedding light on the significant role of key phytohormones in rice’s response to local nitrate signaling.

Several core TFs were identified among OsCBL1-dependent DEGs, suggesting they could be directly or indirectly regulated by OsCBL1. WRKY TFs, known for binding to the W-box cis-acting element of the promoter of target genes, regulate the expression of various gene types and are involved in multiple signaling pathways in plants (Jiang et al., 2017). OsWRKY62, a member of the WRKY family, was found among OsCBL1-dependent DEGs (**Figure 7**; **Supplementary Table S2**). It has been reported that OsWRKY62 is involved in the SA response and disease resistance (Ryu et al., 2006; Liu et al., 2016). In this study, we observed that local nitrate signaling induces the expression of *OsWRKY62* in WT plants, but not in *OsCBL1*-KD plants (**Supplementary Figure S6**), suggesting OsWRKY62 acts downstream of OsCBL1 in the local nitrate signaling pathway. Additionally, OsCBL1 influences members of the bHLH and MYB families, which are also implicated in LR growth regulation in response to local nitrate signaling. Together, these TFs and their associated genes form an OsCBL1-regulated nitrate signaling network, enhancing our understanding of rice’s response mechanisms to local nitrate signaling.

## Data Availability

The datasets presented in this study can be found in online repositories. The names of the repository/repositories and accession number(s) can be found at: NCBI, PRJNA1097064.

## Supplementary material

Supplementary Figure 1 The ECR and LR length of seedling roots of WT and *OsCBL1*-KD. **(A)** The ECR length of WT and *OsCBL1*-KD plants. **(B)** The LR length of WT and *OsCBL1*-KD plants (data are the same as in **Figure 1**). n ≥ 6 biologically independent samples. The error bars represent ± SD. Different letters above bars indicate statistically significant difference between samples (one way ANOVA, P < 0.05).

Supplementary Figure 2 The nitrate content **(A)** and nitrate content ratio **(B)** of WT and *OsCBL1*-KD (data are the same as in **Figure 1**). n = 3 biologically independent samples. The error bars represent ± SD. Different letters above bars indicate statistically significant difference between samples (one way ANOVA, P < 0.05).

Supplementary Figure 3 GO analysis of DEGs in *OsCBL1*-KD. (A) GO analysis of the up-regulated DEGs in *OsCBL1*-KD. (B) GO analysis of the down-regulated DEGs in *OsCBL1*-KD.

Supplementary Figure 4 Venn diagram illustrates the number of GO enrichment of the DEGs of uniquely identified in WT and *OsCBL1*-KD.

Supplementary Figure 5 KEGG analysis of OsCBL1-dependent DEGs. **(A)** KEGG analysis of the up-regulated of OsCBL1-dependent DEGs. **(B)** KEGG analysis of the down-regulated of OsCBL1-dependent DEGs.

Supplementary Figure 6 The expression of key TF in RNA-seq **(A)** and qRT-PCR **(B)**. n =3 biologically independent samples. The error bars represent ± SD. *p < 0.05, and **p < 0.01 compared to the Sp-NaCl (Student’s t-test).

Supplementary Figure 7 Volcano plot of DEGs between Sp-NaNO_3_ and Sp-NaCl in WT.

Supplementary Figure 8 The LR ratio of Sp-NaNO_3_/NaNO_3_ **(A)** and Sp-NaCl/NaCl **(B)** in WT and *OsCBL1*-KD plants. n ≥ 6 biologically independent samples. The error bars represent ± SD. Different letters above bars indicate statistically significant difference between samples (one way ANOVA, P < 0.05).

Supplementary Table 1 The primers used in this article.

Supplementary Table 2 Predicted transcriptional regulatory networks.

## References

[B1] Alvarez J. M. Vidal E. A. Gutiérrez R. A. (2012). Integration of local and systemic signaling pathways for plant N responses. Curr. Opin. Plant Biol. 15, 185–191. doi: 10.1016/j.pbi.2012.03.009 22480431

[B2] Barros J. Serk H. Granlund I. Pesquet E. (2015). The cell biology of lignification in higher plants. Ann. Bot. 115, 1053–1074. doi: 10.1093/aob/mcv046 25878140 PMC4648457

[B3] Bellegarde F. Gojon A. Martin A. (2017). Signals and players in the transcriptional regulation of root responses by local and systemic N signaling in Arabidopsis thaliana. J. Exp. Bot. 68, 2553–2565. doi: 10.1093/jxb/erx062 28369491

[B4] Bolger A. M. Lohse M. Usadel B. (2014). Trimmomatic: a flexible trimmer for Illumina sequence data. Bioinformatics 30, 2114–2120. doi: 10.1093/bioinformatics/btu170 24695404 PMC4103590

[B5] Brunetti C. Fini A. Sebastiani F. Gori A. Tattini M. (2018). Modulation of phytohormone signaling: A primary function of flavonoids in plant-environment interactions. Front. Plant Sci. 9, 1042. doi: 10.3389/fpls.2018.01042 30079075 PMC6062965

[B6] Chen C. Wu Y. Li J. Wang X. Zeng Z. Xu J.. (2023). TBtools-II: A "one for all, all for one" bioinformatics platform for biological big-data mining. Mol. Plant 16, 1733–1742. doi: 10.1016/j.molp.2023.09.010 37740491

[B7] Cheng L. Zhao C. Zhao M. Han Y. Li S. (2022). Lignin Synthesis, Affected by Sucrose in Lotus ( Nelumbo nucifera ) Seedlings, Was Involved in Regulation of Root Formation in the Arabidopsis thanliana. Int. J. Mol. Sci. 23, 2250. doi: 10.3390/ijms23042250 35216366 PMC8875098

[B8] Chu X. Li M. Zhang S. Fan M. Han C. Xiang F.. (2021). HBI1-TCP20 interaction positively regulates the CEPs-mediated systemic nitrate acquisition. J. Integr. Plant Biol. 63, 902–912. doi: 10.1111/jipb.13035 33210841

[B9] Crawford N. M. Glass A. D. M. (1998). Molecular and physiological aspects of nitrate uptake in plants. Trends Plant Sci. 3, 389–395. doi: 10.1016/S1360-1385(98)01311-9

[B10] Dobin A. Davis C. A. Schlesinger F. Drenkow J. Zaleski C. Jha S.. (2013). STAR: ultrafast universal RNA-seq aligner. Bioinformatics 29, 15–21. doi: 10.1093/bioinformatics/bts635 23104886 PMC3530905

[B11] Gao Y. Qi S. Wang Y. (2022). Nitrate signaling and use efficiency in crops. Plant Commun. 3, 13. doi: 10.1016/j.xplc.2022.100353 PMC9483113 35754172

[B12] Guan P. Wang R. Nacry P. Breton G. Crawford N. M. (2014). Nitrate foraging by Arabidopsis roots is mediated by the transcription factor TCP20 through the systemic signaling pathway. Proc. Natl. Acad. Sci. United States America 111, 15267–15272. doi: 10.1073/pnas.1411375111 PMC4210337 25288754

[B13] Ho C. H. Lin S. H. Hu H. C. Tsay Y. F. (2009). CHL1 functions as a nitrate sensor in plants. Cell 138, 1184–1194. doi: 10.1016/j.cell.2009.07.004 19766570

[B14] Hu B. Wang W. Ou S. Tang J. Li H. Che R.. (2015). Variation in NRT1.1B contributes to nitrate-use divergence between rice subspecies. Nat. Genet. 47, 834–838. doi: 10.1038/ng.3337 26053497

[B15] Hu Z. Guo Y. Ying S. Tang Y. Niu J. Wang T.. (2023). OsCBL1 modulates rice nitrogen use efficiency via negative regulation of OsNRT2.2 by OsCCA1. BMC Plant Biol. 23, 502. doi: 10.1186/s12870-023-04520-4 37853334 PMC10583366

[B16] Hu Z. Yuan F. Guo Y. Ying S. Chen J. Zhu D.. (2021). OsCBL1 affects rice seedling growth by modulating nitrate and phosphate responses. Gene 796-797, 145806. doi: 10.1016/j.gene.2021.145806 34197950

[B17] Jiang J. Ma S. Ye N. Jiang M. Cao J. Zhang J. (2017). WRKY transcription factors in plant responses to stresses. J. Integr. Plant Biol. 59, 86–101. doi: 10.1111/jipb.12513 27995748

[B18] Krouk G. Crawford N. M. Coruzzi G. M. Tsay Y. F. (2010a). Nitrate signaling: adaptation to fluctuating environments. Curr. Opin. Plant Biol. 13, 266–273. doi: 10.1016/j.pbi.2009.12.003 20093067

[B19] Krouk G. Lacombe B. Bielach A. Perrine-Walker F. Malinska K. Mounier E.. (2010b). Nitrate-regulated auxin transport by NRT1.1 defines a mechanism for nutrient sensing in plants. Dev. Cell 18, 927–937. doi: 10.1016/j.devcel.2010.05.008 20627075

[B20] Li B. Dewey C. N. (2011). RSEM: accurate transcript quantification from RNA-Seq data with or without a reference genome. BMC Bioinf. 12, 323. doi: 10.1186/1471-2105-12-323 PMC3163565 21816040

[B21] Liu J. Chen X. Liang X. Zhou X. Yang F. Liu J.. (2016). Alternative splicing of rice WRKY62 and WRKY76 transcription factor genes in pathogen defense. Plant Physiol. 171, 1427–1442. doi: 10.1104/pp.15.01921 27208272 PMC4902586

[B22] Liu K. H. Niu Y. Konishi M. Wu Y. Du H. Chung H. S.. (2017). Discovery of nitrate-CPK-NLP signalling in central nutrient-growth networks. Nature 545, 311–316. doi: 10.1038/nature22077 28489820 PMC5823009

[B23] Mounier E. Pervent M. Ljung K. Gojon A. Nacry P. (2014). Auxin-mediated nitrate signalling by NRT1.1 participates in the adaptive response of Arabidopsis root architecture to the spatial heterogeneity of nitrate availability. Plant Cell Environ. 37, 162–174. doi: 10.1111/pce.12143 23731054

[B24] Nacry P. Bouguyon E. Gojon A. (2013). Nitrogen acquisition by roots: physiological and developmental mechanisms ensuring plant adaptation to a fluctuating resource. Plant Soil 370, 1–29. doi: 10.1007/s11104-013-1645-9

[B25] O'Brien J. A. Vega A. Bouguyon E. Krouk G. Gojon A. Coruzzi G.. (2016). Nitrate transport, sensing, and responses in plants. Mol. Plant 9, 837–856. doi: 10.1016/j.molp.2016.05.004 27212387

[B26] Remans T. Nacry P. Pervent M. Filleur S. Gojon A. (2006). The Arabidopsis NRT1.1 transporter participates in the signaling pathway triggering root colonization of nitrate-rich patches. Proc.natl Acad.sci.usa 103, 19206–19211. doi: 10.1073/pnas.0605275103 17148611 PMC1748200

[B27] Riveras E. Alvarez J. M. Vidal E. A. Oses C. Vega A. Gutiérrez R. (2015). The calcium ion is a second messenger in the nitrate signaling pathway of arabidopsis. Plant Physiol. 169, 1397–1404. doi: 10.1104/pp.15.00961 26304850 PMC4587466

[B28] Ruffel S. Krouk G. Ristova D. Shasha D. Birnbaum K. D. Coruzzi G. M. (2011). Nitrogen economics of root foraging: transitive closure of the nitrate-cytokinin relay and distinct systemic signaling for N supply vs. demand. Proc. Natl. Acad. Sci. U.S.A. 108, 18524–18529. doi: 10.1073/pnas.1108684108 22025711 PMC3215050

[B29] Ryu H. S. Han M. Lee S. K. Cho J. I. Ryoo N. Heu S.. (2006). A comprehensive expression analysis of the WRKY gene superfamily in rice plants during defense response. Plant Cell Rep. 25, 836–847. doi: 10.1007/s00299-006-0138-1 16528562

[B30] Sherman B. T. Hao M. Qiu J. Jiao X. Baseler M. W. Lane H. C.. (2022). DAVID: a web server for functional enrichment analysis and functional annotation of gene lists, (2021 update). Nucleic Acids Res. 50, W216–w221. doi: 10.1093/nar/gkac194 35325185 PMC9252805

[B31] Smith S. J. Miller A. J. Fan X. Orsel M. Wells D. M. (2007). Nitrate transport and signalling. J. Exp. Bot. 58, 2297–2306. doi: 10.1093/jxb/erm066 17519352

[B32] Tang R. J. Wang C. Li K. Luan S. (2020). The CBL-CIPK calcium signaling network: unified paradigm from 20 years of discoveries. Trends Plant Sci. 25, 604–617. doi: 10.1016/j.tplants.2020.01.009 32407699

[B33] Tokizawa M. Enomoto T. Chandnani R. Mora-Macías J. Burbridge C. Armenta-Medina A.. (2023). The transcription factors, STOP1 and TCP20, are required for root system architecture alterations in response to nitrate deficiency. Proc. Natl. Acad. Sci. U.S.A. 120, e2300446120. doi: 10.1073/pnas.2300446120 37611056 PMC10469342

[B34] Vanholme R. De Meester B. Ralph J. Boerjan W. (2019). Lignin biosynthesis and its integration into metabolism. Curr. Opin. Biotechnol. 56, 230–239. doi: 10.1016/j.copbio.2019.02.018 30913460

[B35] Varala K. Marshall-Colón A. Cirrone J. Brooks M. D. Pasquino A. V. Léran S.. (2018). Temporal transcriptional logic of dynamic regulatory networks underlying nitrogen signaling and use in plants. Proc. Natl. Acad. Sci. U.S.A. 115, 6494–6499. doi: 10.1073/pnas.1721487115 29769331 PMC6016767

[B36] Wang Y.-Y. Cheng Y.-H. Chen K.-E. Tsay Y.-F. (2018). Nitrate transport, signaling, and use efficiency. Annu. Rev. Plant Biol 69, 85–122. doi: 10.1146/annurev-arplant-042817-040056 29570365

[B37] Wang Q. De Gernier H. Duan X. Xie Y. Geelen D. Hayashi K. I.. (2023). GH3-mediated auxin inactivation attenuates multiple stages of lateral root development. New Phytol. 240, 1900–1912. doi: 10.1111/nph.19284 37743759

[B38] Xu W. Chen Y. Liu B. Li Q. Zhou Y. Li X.. (2023). TaANR1-TaMADS25 module regulates lignin biosynthesis and root development in wheat (Triticum aestivum L.). J. Genet. Genomics 50, 917–920. doi: 10.1016/j.jgg.2023.08.011 37666357

[B39] Yan Y. Wang H. Hamera S. Chen X. Fang R. (2014). miR444a has multiple functions in the rice nitrate-signaling pathway. Plant J. 78, 44–55. doi: 10.1111/tpj.12446 24460537

[B40] Yang J. Deng X. Wang X. Wang J. Du S. Li Y. (2019). The calcium sensor OsCBL1 modulates nitrate signaling to regulate seedling growth in rice. PloS One 14, e0224962. doi: 10.1371/journal.pone.0224962 31697744 PMC6837758

[B41] Zhang H. Forde B. G. (1998). An Arabidopsis MADS box gene that controls nutrient-induced changes in root architecture. Science 279, 407–409. doi: 10.1126/science.279.5349.407 9430595

[B42] Zhang H. Jennings A. Barlow P. W. Forde B. G. (1999). Dual pathways for regulation of root branching by nitrate. Proc. Natl. Acad. Sci. U.S.A. 96, 6529–6534. doi: 10.1073/pnas.96.11.6529 10339622 PMC26916

[B43] Zhang B. Zhu X. Chen Z. Zhang H. Huang J. Huang J. (2023). RiceTFtarget: A rice transcription factor-target prediction server based on coexpression and machine learning. Plant Physiol. 193, 190–194. doi: 10.1093/plphys/kiad332 37294915

