# Supplementary material for: OsCBL1 mediates rice response to local nitrate signaling: insights into regulatory networks and gene expression
Source: Front Plant Sci. 2024 Sep 13;15:1418119. doi: 10.3389/fpls.2024.1418119 (PMC11427294; doi:10.3389/fpls.2024.1418119)
Supplement: Supplementary Figure 1 — The ECR and LR length of seedling roots of WT and OsCBL1-KD. (A) The ECR length of WT and OsCBL1-KD plants. (B) The LR length of WT and OsCBL1-KD plants (data are the same as in Figure 1 ). n ≥ 6 biologically independent samples. The error bars represent ± SD. Different letters above bars indicate statistically significant difference between samples (one way ANOVA, P < 0.05). [file DataSheet1.pdf]

Supplementary Material

Title: OsCBL1 Mediates Rice Response to Local Nitrate Signaling: Insights into Regulatory Networks and Gene Expression

Zhao Hu[1], Dongchen He[1], Xiaojue Peng[1*], Jing Yang[1*]

1. College of Life Science, Nanchang University, Nanchang, China

Corresponding author: Jing Yang, Xiaojue Peng

Email: yangjing@ncu.edu.cn, xiaojuepeng@ncu.edu.cn

Supplementary Figures and Tables

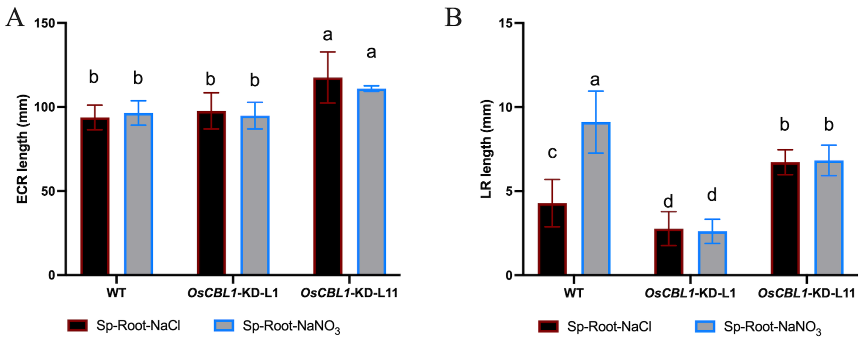

Supplementary Figure 1. The ECR and LR length of seedling roots of WT and *OsCBL1*-KD. **(A)** The ECR length of WT and *OsCBL1*-KD plants. **(B)** The LR length of WT and *OsCBL1*-KD plants (data are the same as in Figure 1). n ≥ 6 biologically independent samples. The error bars represent ± SD. Different letters above bars indicate statistically significant difference between samples (one way ANOVA, P < 0.05).

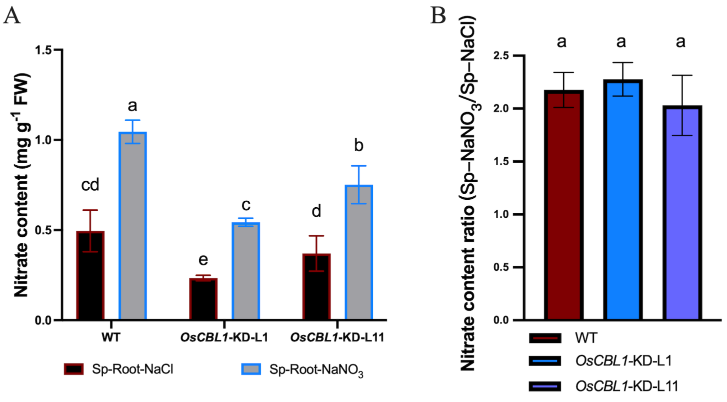

Supplementary Figure 2. The nitrate content **(A)** and nitrate content ratio **(B)** of WT and *OsCBL1*-KD (data are the same as in Figure 1). n = 3 biologically independent samples. The error bars represent ± SD. Different letters above bars indicate statistically significant difference between samples (one way ANOVA, P < 0.05).

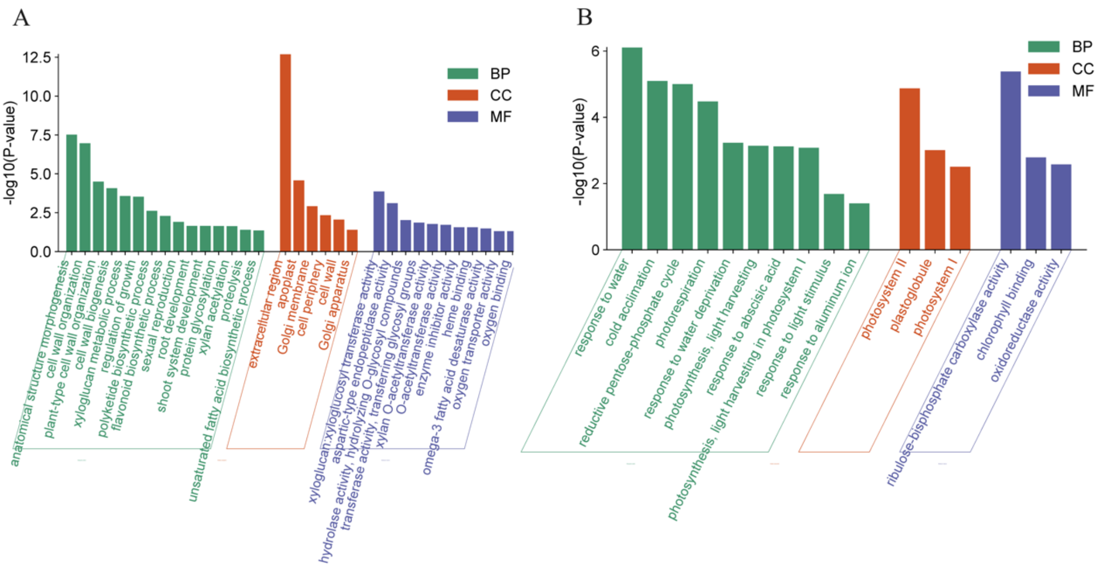

Supplementary Figure 3. GO analysis of DEGs in *OsCBL1*-KD. **(A)** GO analysis of the up-regulated DEGs in *OsCBL1*-KD. **(B)** GO analysis of the down-regulated DEGs in *OsCBL1*-KD.

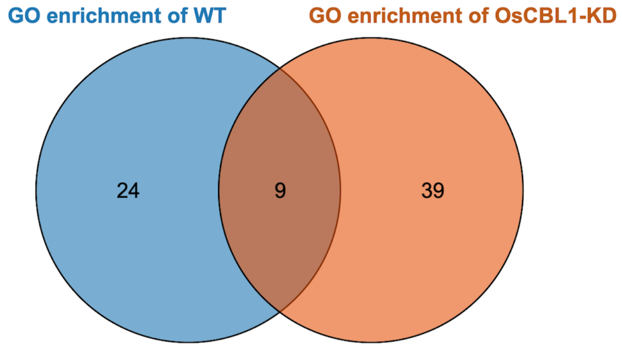

Supplementary Figure 4. Venn diagram illustrates the number of GO enrichment of the DEGs of uniquely identified in WT and *OsCBL1*-KD.

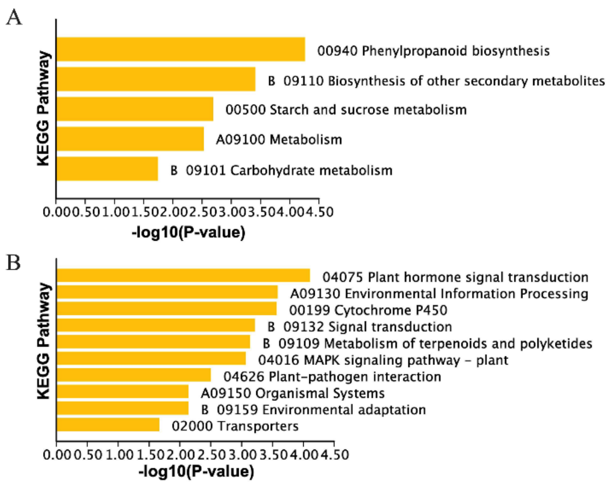

Supplementary Figure 5. KEGG analysis of OsCBL1-dependent DEGs. **(A)** KEGG analysis of the up-regulated of OsCBL1-dependent DEGs. **(B)** KEGG analysis of the down-regulated of OsCBL1-dependent DEGs.

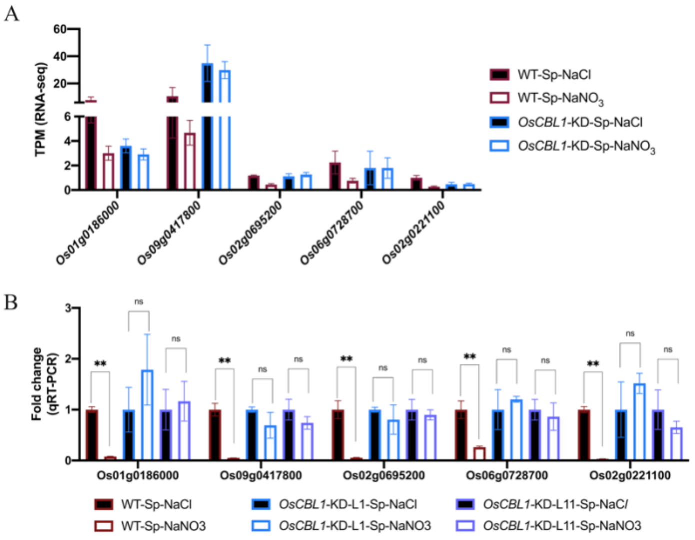

Supplementary Figure 6. The expression of key TF in RNA-seq **(A)** and qRT-PCR **(B)**. n =3 biologically independent samples. The error bars represent ± SD. $*p < 0.05$, and $**p < 0.01$ compared to the Sp-NaCl (Student's t-test).

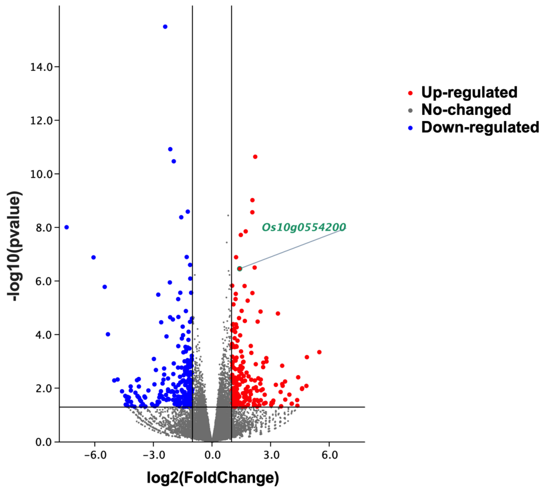

Supplementary Figure 7. Volcano plot of DEGs between Sp-$NaNO_3$ and Sp-NaCl in WT.

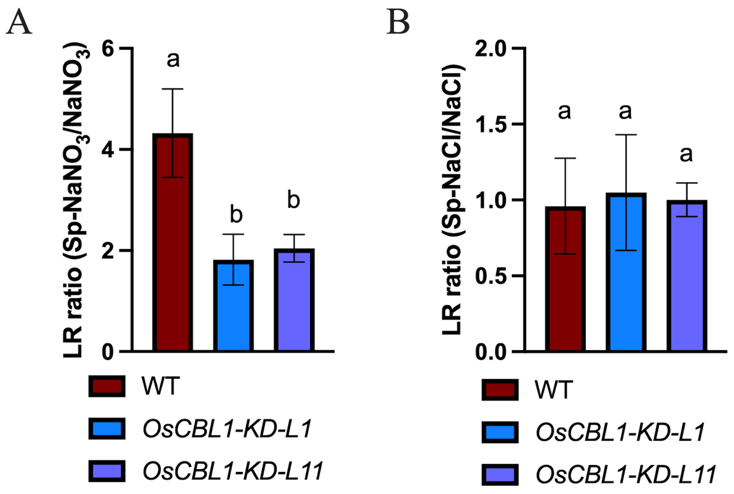

Supplementary Figure 8. The LR ratio of Sp-$NaNO_3$/$NaNO_3$ **(A)** and Sp-NaCl/NaCl **(B)** in WT and *OsCBL1*-KD plants. n ⩾ 6 biologically independent samples. The error bars represent ± SD. Different letters above bars indicate statistically significant difference between samples (one way ANOVA, $P < 0.05$).

Supplementary Table 1: The primers used in this article.

Gene Name	Gene ID	Forward primer	Revers primer	Primer efficiency
OsPrx80	Os06g0490400	GCTCTACAACT TCACCGGCA	AGTAGCTGGTG TCGAACGTC	103.33%
OsPrx41	Os03g0339300	GGCTTGTCCGG TTTATTGCC	GGCAGCAATGA CAAGAGTACG	106.18%
OsPrx39	Os03g0234900	TTCAAGAGCTT CGCCAGGTC	CAGTCCTTCGA GCTAACGGG	100.94%

OsBGLU29	Os09g0490400	CTGGACGTTCATGGACTGCT	GTAGCTGGACTGCTTGTGGT	101.73%
OsBGLU3	Os01g0813800	TCCTGAATGCGACCAGGAAC	TCGAAATCCACGCGGTAGAG	96.94%
NA	Os07g0156467	ATCAAGGATGTGGAGACCGC	GCTTGTATCGCTTCAGCACG	101.44%
OsPrx7	Os07g0157000	TGAAGCGATACAAGCGGAGG	CCCCTAAAACGCGACAGGAT	100.92%
OsPrx121	Os09g0323700	CCTGCGACCAGAACCTCTAC	GAACTTGCTGCAGACCTTGC	109.93%
NA	Os10g0566200	TTGAGAGCCATCTTGAGCCG	GTGTCTCCTAGCGTCTGCTG	104.30%
OsPR1b	Os07g0125000	TGCGTATGCATGATCAGTGTG	GCGTCATACATTAAAATACGGAGGG	106.67%
NA	Os07g0125600	TACGACTACGCCAGCAACAG	TACGGCCTCTGGTTTTGGAC	104.23%
NA	Os07g0127700	TCAGCTGCAACTATTCCCCG	ACACACACAATCCGGCTACA	109.03%
NA	Os07g0126401	GAGAACCTGTCGTGGAACCC	TGTTGCTGGCGTAGTCGTAG	105.03%
OsGH3.1	Os01g0785400	GCTCATGGACTACGCGATCA	TGAAGTACTTGCCCTGCACC	109.53%
OsPIL14	Os07g0143200	GGCACCGGCAATATGTGAAG	CACCAGATGCATGTCCCTCA	109.37%
NA	Os11g0514500	TGTCGGATCAACTGGGTGGA	CCATCTCCCAAGGTGTGTGG	107.10%
OsWRKY10	Os01g0186000	TGATCAGTATGGCGTGTCGT	CTTCCCGTACTTTCGCCACT	106.30%
OsWRKY62	Os09g0417800	CACTCGACCTGACGAACCAA	CGTTCTTGAGCACTTGCACC	105.46%
OsMYB58/63a	Os02g0695200	TCGGCCACTGCACTATCATC	TGTTTGACGGCTTGACCCTT	101.28%
OsEPR1	Os06g0728700	AGCGCAAATTCAGCGTTTGT	AAGCAACGTTCGTGCTCCTA	103.26%
OsbHLH029	Os02g0221100	CCAAGAACCGAGCACTCCAA	ACGGCTAGCCATTGGTGATT	107.91%
OsNRT1.1B	Os10g0554200	GGCAGGCTCGACTACTTCTA	AGGCGCTTCTCCTTGTAGAC	104.58%
OsACTIN1	Os03g0718100	ACCATTGGTGCTGAGCGTTT	CGCAGCTTCCATTCCTATGAA	108.00%

Supplementary Table 2: Predicted transcriptional regulatory networks.

TF	Target
WRKY10	Os08g0403300
WRKY10	Os05g0324700
WRKY10	Os01g0721200
WRKY10	Os03g0234900
WRKY10	Os03g0644600
WRKY10	Os10g0393800
WRKY10	Os10g0562900
WRKY10	Os12g0478200
WRKY62	Os03g0339300
WRKY62	Os05g0161500
WRKY62	Os05g0324700
WRKY62	Os04g0584100
WRKY62	Os10g0527400
WRKY62	Os05g0578100
WRKY62	Os12g0555000
WRKY62	Os02g0193200
WRKY62	Os07g0418500
WRKY62	Os10g0562900
WRKY62	Os12g0603800
WRKY62	Os03g0664800
MYB58/63a	Os03g0294100
MYB58/63a	Os07g0106700
EPR1	Os12g0633600
EPR1	Os06g0661200
EPR1	Os02g0622500
bHLH029	Os06g0140300
bHLH029	Os04g0541700
bHLH029	Os01g0878400
bHLH029	Os07g0583500
bHLH029	Os06g0136300
